# Epidemiological Trends and Projection of Liver Cancer Due to Nonalcoholic Steatohepatitis Among People Aged 55 Years and Older in China From 1990 to 2030: An Analysis of the Global Burden of Disease Study 2021

**DOI:** 10.14309/ctg.0000000000000872

**Published:** 2025-06-11

**Authors:** Xi Yang, Xiaodan Yin, Huiqi Wu, Qiaomei Li, Yang Shen

**Affiliations:** 1 Department of General Surgery, the Affiliated Shuyang Hospital of Xuzhou Medical University, Suqian, Jiangsu, China;; 2 Department of Neurology, the Third Affiliated Hospital of Naval Medical University, Shanghai, China;; 3 Department of Critical Care Medicine, the Third Affiliated Hospital of Naval Medical University, Shanghai, China;; 4 Department of Biliary Medicine, the Third Affiliated Hospital of Naval Medical University, Shanghai, China.

**Keywords:** liver cancer, metabolic dysfunction-associated steatohepatitis, global burden of disease

## Abstract

**INTRODUCTION:**

The aim of this study was to characterize the temporal trends of liver cancer due to metabolic dysfunction-associated steatohepatitis (LCDMDS) burden in China during 1990–2021; evaluate their age, period, and cohort effects; and predict the disease burden for the next 9 years.

**METHODS:**

Data were obtained from the Global Burden of Disease 2021 study. Joinpoint regression model was used to estimate the annual percentage change (APC) and the average APC of LCDMDS incidence and death rates, and the age-period-cohort analysis was used to estimate the age, period, and cohort effects. We extended the autoregressive integrated moving average (ARIMA) model to predict the disease burden of LCDMDS in 2022–2030.

**RESULTS:**

From 1990 to 2021, the number of incidence cases, incidence rates, number of deaths, and death rates of LCDMDS among the Chinese population aged 55 years and older all increased significantly. The number of incidence cases rose from 2,763 to 9,018, the incidence rate increased from 1.93 per 100,000 to 2.38 per 100,000, the number of deaths increased from 2,942 to 8,721, and the death rate rose from 2.05 per 100,000 to 2.30 per 100,000. The average APCs of the incidence rate and death rate were 0.72% (*P* < 0.05) and 0.42% (*P* > 0.05), respectively. Taking the average levels of age, period, and cohort as reference, the relative risks of LCDMDS incidence and death first increased and then decreased with age, increased over time, and decreased with the development of the birth cohort. The prediction results of the ARIMA model showed that the number of incidence cases and deaths among the Chinese population aged 55 years and older will continue to increase from 2022 to 2030, whereas the incidence rate and death rate will show slight changes.

**DISCUSSION:**

This study comprehensively explored the temporal trends of LCDMDS burden among Chinese aged 55 years and older from 1990 to 2021, revealing significant increases in incidence and mortality, as well as age, period, and cohort effects. ARIMA model projections show that the LCDMDS burden will continue to rise despite minor rate changes. Thus, immediate interventions such as early detection, public—health awareness—raising, and further research are urgently needed to relieve the LCDMDS burden in China.

## INTRODUCTION

Liver cancer is one of the most lethal malignant tumors globally, and its high morbidity and mortality impose a heavy economic and psychological burden on patients and their families. The disease burden associated with liver cancer is extremely heavy and its incidence is increasing (1), and it has become a major public health problem worldwide (2,3). In 2021, there were 529,000 liver cancer cases, 484,000 deaths, and 12,900,000 disability-adjusted life years (DALYs) in the world (4). This is an increase of 114.3%, 102.5% and 70.6%, respectively, compared with 1990 (5). China ranks among the countries with the highest burden of liver cancer regarding incidence, deaths, and DALYs globally (6).

The incidence of liver cancer of different etiologies has been gradually increasing over the last 30 years (1). Hepatitis B virus and hepatitis C virus are the leading causes of liver cancer, accounting for a large proportion of liver cancer cases and deaths (1,7), but their importance is likely to decrease in the future (8). In addition, alcohol consumption and metabolic dysfunction-associated steatohepatitis (MASH) are important factors. MASH is a metabolic stress liver injury closely related to insulin resistance and genetic susceptibility (9). It is characterized by hepatocellular steatosis, inflammation, and hepatocellular damage as the main pathological features and may progress to liver fibrosis, cirrhosis, and even liver cancer (10). Globally, the incidence of MASH is on the rise and it is associated with metabolic syndromes such as obesity and diabetes (9), and the prevalence of MASH is expected to continue to rise in the future with the increase of these diseases (11,12). There is growing concern about the impact of MASH on liver cancer (13). 8.5% of global liver cancer deaths in 2021 were attributed to MASH, making it the fourth leading cause of death from liver cancer (5), and the contribution of MASH to the cause of death from liver cancer is rising (14). Liver cancer due to MASH (LCDMDS), an important type of liver cancer, has been increasing in incidence in recent years. In 2019, the global number of incidence, prevalence, and deaths of LCDMDS has reached 36.3 thousand, 46.8 thousand, and 34.7 thousand (15). LCDMDS poses a serious threat to public health.

Aging is an important challenge that the world is facing. Aging is a major risk factor for cancer development (16), and LCDMDS occurs mostly in the elderly population (15,17). The prevalence of steatohepatitis with advanced fibrosis is much higher in elderly patients with steatohepatitis than in nonelderly patients (18). The incidence and mortality of LCDMDS in individuals aged older than 55 years increased significantly from 2010 to 2019 (15). Although existing studies have examined the global and regional trends of liver cancer and LCDMDS over the past 3 decades, there is a relative lack of studies with middle-aged and elderly individuals. China, the world's most populous country, is also one of the regions with the deepest degree of population aging, and with the continuous economic development in the past 3 decades, the lifestyle has changed dramatically, and the incidence of various metabolic diseases has been increasing. Therefore, it is necessary to assess the epidemiological changes in the disease burden of LCDMDS in middle-aged and elderly people in China.

In summary, we used data from Global Burden of Disease (GBD) 2021 to analyze the changes in the burden of LCDMDS in Chinese people aged 55 years and older over the period 1990–2021. Joinpoint regression was used to estimate the trends of incidence rates and death rates over time. An age-time-cohort model was used to assess the relationship between the risk of incidence and death from LCDMDS and age, time period, and birth cohort. Finally, autoregressive integrated moving average (ARIMA) model was used to predict the burden of LCDMDS from 2022 to 2030. This study not only improves the knowledge of the developmental pattern of LCDMDS in specific populations and forms a comprehensive cognitive system but also provides a basis for public health decision making, helps to formulate prevention and control strategies, reduces the burden of disease, and lays the foundation for subsequent research.

## METHODS

### Data sources

Data on the burden of LCDMDS in China from 1990 to 2021 were retrieved from the GBD 2021 public database (https://ghdx.healthdata.org/). The GBD 2021 offers comprehensive data on 369 diseases and injuries, including incidence, prevalence, mortality, years lived with disability, years of life lost, and DALYs, along with their corresponding 95% uncertainty intervals (UIs). These data span both sexes, 87 risk factors, and 204 countries and territories. It encompasses a vast array of 86,249 data sources, ranging from censuses, household surveys, civil registration and vital statistics, disease registries, healthcare utilization, air pollution monitoring, satellite imaging, disease notifications, and more. The GBD data were constructed based on all available existing epidemiological data (both published and unpublished) and routinely collected data (e.g., hospitalization and medical care data, etc.) by applying cause-of-death pooled models, spatiotemporal Gaussian regression models, and Bayesian regression. Numerous research studies on a spectrum of diseases using Chinese GBD data have been executed, substantiating the data's dependability and its representation of the general population (19–21). As the research did not involve direct participation of human subjects, ethical approval was not mandated.

### Joinpoint regression model

The Joinpoint regression model will be used to analyze the trends in the incidence rates and death rates of LCDMDS and to identify significant joinpoints, thereby delineating the temporal patterns and notable shifts in the incidence rates and death rates (22). Based on the temporal characteristics of the disease distribution, the Joinpoint regression model will build a segmented regression model with trend fitting and optimization for each segmented data point. Joinpoint regression modeling uses Monte Carlo permutation test to determine the best-fitting model. The equation of the log-linear model is: *Ε* [*y*|*x*] = $e^{\beta_{0}+\beta_{1}Χ+\delta_{1}{\left(x-\tau_{1}\right)}^{+}+\ldots +\delta_{k}{\left(x-\tau_{k}\right)}^{+}}$, where *y* is disease incidence rate, *x* is year, *β*_1_ is regression coefficient, *k* is the number of joinpoints, the $\tau_{k}$ are the unknown joinpoints, and a^+^ = a for a > 0 and 0 otherwise. Model results can be summarized using the metrics of annual percentage change (APC) and average APC (AAPC) (23). The APC is calculated as APC = $\left[\frac{y_{x+1}-y_{x}}{y_{x}}\right]\times$100% = ($e^{\beta_{1}}$ − 1) × 100%, evaluating the trend of independent intervals of piecewise functions. APC > 0, incidence rate is increasing over time; APC < 0, incidence rate is decreasing over time. The AAPC is calculated as AAPC = ($e^{\sum \omega_{i}\beta_{i}/\sum \omega_{i}}-1$) ×100%, assessing the average trend over the entire study interval, where $\omega_{i}$ is the interval year of each segmented function, and $\beta_{i}$ is the regression coefficient corresponding to each interval.

### Age-period-cohort model

The age-period-cohort model is a common epidemiological analysis tool and is widely used to analyze chronic disease incidence trends. It is based on the Poisson distribution, which decomposes the target variable in 3 dimensions: age, period, and cohort, so as to better analyze the risk of disease onset in these 3 dimensions (24). The age effect is the risk of outcomes across age groups. The period effect refers to the effect of time change on outcomes across all age groups. Cohort effects refer to changes in outcomes between participants in the same birth cohort. The log-linear regression model is expressed as follows: log(Yi) = μ + α × agei + β × periodi + γ × cohorti + ε, where Yi is the LCDMDS incidence rates and death rates; α, β and γ are the coefficients of age, period, and cohort, respectively; μ is the intercept; and ε is the residual of model. Owing to the linear relationship between age, period, and cohort, the intrinsic estimator method (25) was used to obtain the net effect of the 3 dimensions to avoid biased results.

### Autoregressive integrated moving average model

The ARIMA model consists of autoregressive (AR) model, moving average (MA) model, and difference process (I), and the basic form of the model is ARIMA (*p*, *d*, *q*), where *p* is the order of autoregression, *d* is the order of difference, and *q* is the order of MA (26,27). The ARIMA model is a common model for time series analysis and forecasting by using historical information from the data itself to predict future trends. This study transformed the historical data on the incidence and mortality of LCDMDS among the Chinese population aged 55 years and older into time series and then predicted the burden of LCDMDS in China from 2022 to 2030. The AR model describes the relationship between current and historical values and is based on historical values to predict future values. The MA model uses the error associated with the prediction at the previous time step to predict the variable at the subsequent time step, focusing on historical information about the prediction error. I is the differencing process, which is mainly used to deal with nonstationary series, and d is the order of the differencing, which is *d* = 0 if the time series itself is stationary. The equation is expressed as Yt = $\varphi_{1}$ Yt − 1 + $\varphi_{2}$ Yt−2 + … + $\varphi_{p}$ Yt−*p* + $e_{t}$ - $\theta_{1}e_{t-1}$ … -$\theta_{q}e_{t-q}$, where ($\varphi_{1}$ Yt−1 + $\varphi_{2}$ Yt−2 + … + $\varphi_{p}$ Yt−*p* + $e_{t}$ ) is the AR model part, ($e_{t}$ - $\theta_{1}e_{t-1}$ - …-$\theta_{q}e_{t-q})$ is the MA model part, Yt−p is the observed value at the period of (*t* − *p*), *p* and *q* represent the model order of AR and MA, and $e_{t}$ is the random error at the period of *t*.

### Statistical analysis

In GBD 2021, the incidence rate and death rate of LCDMDS were defined as the number of cases and deaths of LCDMDS per 100,000 total population. The age-standardized incidence rate (ASIR) and age-standardized mortality rate (ASMR) are calculated using the Chinese standard population as the reference population. Joinpoint analysis was run in the Joinpoint regression 4.9 software (Statistical Research and Applications Branch, National Cancer Institute, USA). To run the age- period-cohort model, we divided the data series into consecutive 5-year intervals from 1990 to 2021, with the 2020–2021 interval analyzed as a separate interval. We analyzed the population of middle-aged and older adults aged 55 years and older who developed LCDMDS. Ages were also divided into 5-year age groups ranging from 55 to 59 years to 95 years or older. Calculate the birth cohort by subtracting age from period. We collated 15 cohorts covering subjects born from 1895–1899 to 1965–1969. The mean level of age, period, and cohort was selected as the reference groups (28). Relative risk (RR) values for each age, period, and cohort represent independent risk compared with the reference group [Exp (*α* – *α*_mean_), Exp (*β* – *β*_mean_), Exp (*γ* – *γ*_mean_)]. Age-period-cohort model was created in Stata 14.0 software (StataCorp LP, TX). In the ARIMA modeling procedure, we initially applied the difference method to achieve stationarity in the time-series data set. The optimal model was identified using the *auto.arima*() function, which is based on minimizing the Akaike Information Criterion and Bayesian Information Criterion (29). To assess the normality of the residuals, we used Q-Q plots, autocorrelation function plots, and partial autocorrelation function plots. Subsequently, the Ljung-Box test was conducted to determine if the residuals were white noise. All ARIMA analyses and plot representations were performed using R software version 4.4.1 (R Development Core Team), leveraging the “forecast” and “ggplot2” packages. Statistical significance was determined using a 2-tailed test with *P* < 0.05.

## RESULTS

**Figure 1.**
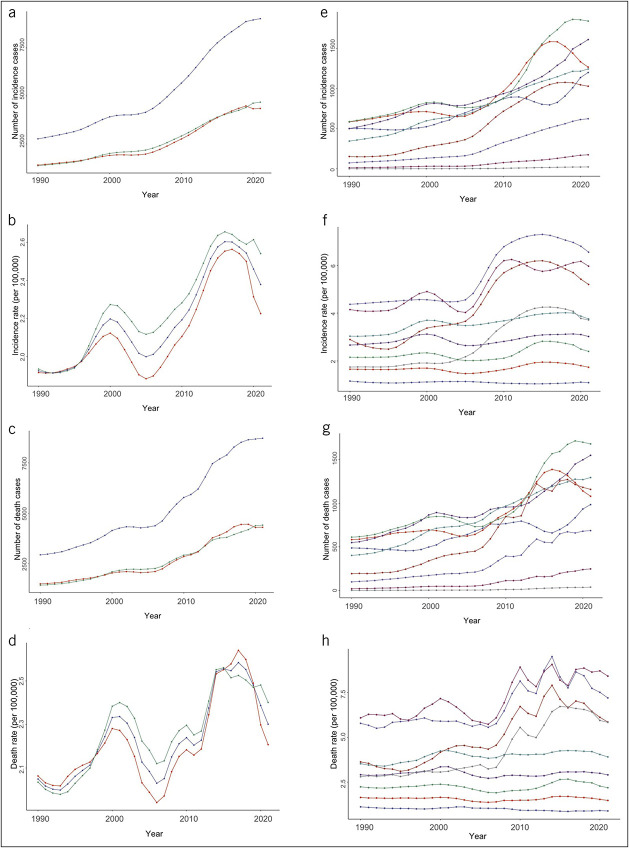
Number of incidence/death cases and incidence/death rates of LCDMDS in Chinese population aged 55 years or older from 1990 to 2021. (**a**) Number of incidence cases in LCDMDS by sex groups. (**b**) Incidence rates in LCDMDS by sex groups. (**c**) Number of death cases in LCDMDS by sex groups. (**d**) Death rates in LCDMDS by sex groups. (**e**) Number of incidence cases in LCDMDS by age groups. (**f**) Incidence rates in LCDMDS by age groups. (**g**) Number of death cases in LCDMDS by age groups. (**h**) Death rates in LCDMDS by age groups. LCDMDS, liver cancer due to metabolic dysfunction-associated steatohepatitis.

Between 1990 and 2021 in China, among individuals aged 55 years or older, the number of LCDMDS incidence cases, incidence rates, deaths, and death rates all increased. The number of incidence cases rose from 2,763 to 9,018, a 226.38% increase. The incidence rate increased from 1.93 per 100,000 to 2.38 per 100,000, a 23.32% rise. The number of death cases grew from 2,942 to 8,721, an increase of 196.43%. The death rate increased from 2.05 per 100,000 to 2.30 per 100,000, a 12.20% growth. In addition, the ASIR and ASMR showed a slight upward trend. The ASIR increased from 0.48 per 100,000 to 0.54 per 100,000 (a 12.20% increase), and the ASMR rose from 0.50 per 100,000 to 0.51 per 100,000 (a 12.20% increase). The burden of incidence and death in LCDMDS increased in all indicators for both men and women, with similar trends. The number of incidence and death significantly increased in different age groups, with an increase of approximately 1 - fold or more. The number of incidence and death first increased and then decreased with age, peaking in the 65–69 years old age group. Incidence rates and death rates followed similar patterns (Table 1). The trends in the LCDMDS burden over the years are presented in Figure 1.

**Table 1.** Number of incidence/death cases and incidence/death rates of LCDMDS in Chinese population aged 55 yr or older by sex and age groups, 1990–2021

Group	1990				2021			
	Number of incidence (95% UI)	Incidence rate, per 100,000 (95% CI)	Number of deaths (95% UI)	Mortality rate, per 100,000 (95% CI)	Number of incidence (95% UI)	Incidence rate, per 100,000 (95% CI)	Number of deaths (95% UI)	Mortality rate, per 100,000 (95% CI)
Total	2,763 (2,146–3,514)	1.93 (1.50–2.45)	2,942 (2,307–3,701)	2.05 (1.61–2.58)	9,018 (6,837–11,654)	2.38 (1.80–3.08)	8,721 (6,611–11,213)	2.30 (1.74–2.96)
Sex								
Male	1,367 (1,004–1,809)	1.93 (1.42–2.56)	1,438 (1,061–1,892)	2.04 (1.50–2.68)	4,673 (3,232–6,611)	2.54 (1.76–3.60)	4,413 (3,071–6,266)	2.40 (1.67–3.41)
Female	1,397 (1,034–1,806)	1.92 (1.42–2.48)	1,504 (1,123–1,946)	2.06 (1.54–2.67)	4,345 (3,272–5,819)	2.23 (1.68–2.98)	4,308 (3,220–5,757)	2.21 (1.65–2.95)
Age								
55–59	502 (354–689)	1.16 (0.82–1.59)	487 (343–668)	1.12 (0.79–1.54)	1,201 (793–1,744)	1.09 (0.72–1.59)	985 (651–1,430)	0.90 (0.59–1.3)
60–64	585 (414–805)	1.65 (1.17–2.28)	583 (412–801)	1.65 (1.16–2.27)	1,268 (841–1,851)	1.74 (1.15–2.54)	1,080 (715–1,573)	1.48 (0.98–2.16)
65–69	588 (406–790)	2.16 (1.49–2.90)	611 (424–821)	2.24 (1.55–3.01)	1,842 (1,245–2,560)	2.40 (1.62–3.34)	1,681 (1,140–2,336)	2.19 (1.49–3.05)
70–74	502 (358–670)	2.67 (1.90–3.56)	549 (390–732)	2.92 (2.07–3.89)	1,611 (1,111–2,176)	3.02 (2.08–4.08)	1,550 (1,068–2,084)	2.91 (2.00–3.91)
75–79	346 (247–470)	3.04 (2.17–4.13)	402 (287–542)	3.53 (2.52–4.76)	1,245 (875–1,694)	3.76 (2.64–5.11)	1,295 (911–1,761)	3.91 (2.75–5.32)
80–84	154 (109–210)	2.90 (2.06–3.97)	192 (137–260)	3.63 (2.59–4.92)	1,030 (717–1,420)	5.20 (3.62–7.18)	1,159 (815–1,601)	5.86 (4.12–8.09)
85–89	74 (53–97)	4.37 (3.15–5.75)	98 (70–129)	5.79 (4.15–7.65)	624 (434–833)	6.55 (4.56–8.75)	686 (475–921)	7.20 (4.99–9.67)
90–94	13 (9–18)	4.15 (2.79–5.77)	19 (13–26)	6.09 (4.10–8.45)	175 (113–246)	5.97 (3.86–8.38)	247 (161–347)	8.42 (5.48–11.82)
≥95	1 (0–1)	1.74 (1.09–2.64)	1 (1–2)	2.80 (1.75–4.24)	24 (14–37)	3.71 (2.20–5.76)	37 (22–58)	5.84 (3.46–9.06)

CI, confidence interval; LCDMDS, liver cancer due to metabolic dysfunction-associated steatohepatitis; UI, uncertainty interval.

### The temporal trend of LCDMDS

According to the results of Joinpoint regression analysis, from 1990 to 2021, the incidence rate of LCDMDS among the Chinese population aged 55 years or older exhibited a significant upward trend (APC 0.72%, *P* < 0.05). Specifically, it increased slightly from 1990 to 1995 (APC 0.17%), rose from 1995 to 2000 (APC 2.84%, *P* < 0.05), decreased from 2000 to 2005 (APC −2.77%, *P* < 0.05), increased from 2005 to 2011 (APC 2.08%, *P* < 0.05), increased from 2011 to 2015 (APC 3.77%, *P* < 0.05), decreased slightly from 2015 to 2018 (APC −0.02%), and decreased from 2018 to 2021 (APC −2.73%, *P* < 0.05) (Table 2). The incidence rates increased in both men and women, and the AAPC of the incidence rate in men was higher than that in women (0.94% vs 0.51%, both *P* < 0.05). Among all age groups, except for the 55–59 years group, the AAPC of each group was > 0 (*P* < 0.05), indicating an actual increase in the LCDMDS incidence in each age group (Table 2). Meanwhile, from 1990 to 2021, the death rate of LCDMDS did not show an overall increase (APC 0.42%, *P* > 0.05). It decreased slightly from 1990 to 1994 (APC -0.45%), increased from 1994 to 2001 (APC 2.39%, *P* < 0.05), decreased from 2001 to 2006 (APC −3.03%, *P* < 0.05), increased from 2006 to 2017 (APC 2.39%, *P* < 0.05), and decreased from 2017 to 2021 (APC −3.06%, *P* < 0.05) (Table 3). The death rate in men increased significantly (APC 0.60%, *P* < 0.05), whereas that in women did not (APC 0.38%, *P* > 0.05). The death rates in the 55–59, 60–64, and 65–69 years age groups decreased significantly (*P* < 0.05), whereas the death rate in the age group of 80 years and older increased significantly (Table 3).

**Table 2.** Joinpoint regression analysis on the incidence rates of LCDMDS in Chinese population aged 55 yr or older, 1990 to 2021

Group	Total study period AAPC (%, 95% CI)	Trend 1		Trend 2		Trend 3		Trend 4		Trend 5		Trend 6		Trend 7	
		Years	APC (%, 95% CI)	Years	APC (%, 95% CI)	Years	APC (%, 95% CI)	Years	APC (%, 95% CI)	Years	APC (%, 95% CI)	Years	APC (%, 95% CI)	Years	APC (%, 95% CI)
Total	0.72* (0.61 to 0.82)	1990–1995	0.17 (−0.01 to 0.35)	1995–2000	2.84* (2.6 to 3.08)	2000–2005	−2.27* (−2.48 to −2.06)	2005–2011	2.08* (1.92 to 2.24)	2011–2015	3.77* (3.39 to 4.15)	2015–2018	−0.02 (−0.78 to 0.74)	2018–2021	−2.73* (−3.18 to −2.29)
Sex															
Male	0.94* (0.82 to 1.07)	1990–1995	0.01 (−0.27 to 0.29)	1995–2000	3.65* (3.27 to 4.03)	2000–2005	−1.79* (−2.13 to −1.44)	2005–2011	1.69* (1.45 to 1.93)	2011–2015	3.47* (2.89 to 4.06)	2015–2021	−0.59* (−0.84 to −0.35)		
Female	0.51* (0.33 to 0.68)	1990–1995	0.35* (0.04 to 0.66)	1995–2000	2.02* (1.61 to 2.44)	2000–2005	−2.79* (−3.15 to −2.44)	2005–2011	2.51* (2.24 to 2.78)	2011–2015	4.13* (3.53 to 4.74)	2015–2018	0.41 (−0.84 to 1.66)	2018–2021	−4.64* (−5.4 to −3.88)
Age															
55–59	−0.12 (−0.32 to 0.07)	1990–1995	−1.42* (−1.76 to −1.08)	1995–2002	0.88* (0.61 to 1.14)	2002–2005	0.12 (−1.34 to 1.6)	2005–2011	−1.29* (−1.63 to −0.96)	2011–2015	−0.36 (−1.12 to 0.41)	2015–2021	1.02* (0.73 to 1.31)		
60–64	0.19* (0.03 to 0.34)	1990–1995	−0.2 (−0.44 to 0.05)	1995–2000	0.89* (0.56 to 1.23)	2000–2005	−3.16* (−3.47 to −2.84)	2005–2009	1.97* (1.44 to 2.5)	2009–2014	4.16* (3.8 to 4.53)	2014–2017	0.27 (−0.86 to 1.41)	2017–2021	−2.62* (−3.02 to −2.22)
65–69	0.33* (0.21 to 0.46)	1990–1995	0.07 (−0.15 to 0.29)	1995–2000	1.82* (1.51 to 2.12)	2000–2005	−3.29* (−3.57 to −3.01)	2005–2010	0.97* (0.68 to 1.25)	2010–2015	6.54* (6.23 to 6.85)	2015–2019	−1.82* (−2.29 to −1.34)	2019–2021	−5.57* (−6.66 to −4.47)
70–74	0.42* (0.33 to 0.51)	1990–1995	0.74* (0.59 to 0.89)	1995–2000	2.75* (2.55 to 2.95)	2000–2005	−3.66* (−3.85 to −3.47)	2005–2008	1.02* (0.42 to 1.62)	2008–2014	2.06* (1.92 to 2.2)	2014–2019	0.51* (0.3 to 0.72)	2019–2021	−1.75* (−2.47 to −1.02)
75–79	0.70* (0.59 to 0.81)	1990–1995	0.42* (0.25 to 0.59)	1995–2000	3.92* (3.69 to 4.15)	2000–2005	−1.53* (−1.75 to −1.31)	2005–2008	1.01* (0.31 to 1.72)	2008–2015	1.57* (1.45 to 1.69)	2015–2019	0.21 (−0.16 to 0.58)	2019–2021	−3.32* (−4.1 to −2.53)
80–84	2.00* (1.73 to 2.27)	1990–1995	−3.02* (−3.61 to −2.42)	1995–2000	6.92* (6 to 7.85)	2000–2005	1.23* (0.41 to 2.06)	2005–2010	10.05* (9.22 to 10.89)	2010–2016	1.14* (0.57 to 1.71)	2016–2021	−3.45* (−4.03 to −2.87)		
85–89	1.37* (1.24 to 1.49)	1990–1998	0.53* (0.36 to 0.7)	1998–2005	−0.21 (−0.45 to 0.04)	2005–2010	8.99* (8.52 to 9.45)	2010–2015	1.32* (0.88 to 1.77)	2015–2021	−1.70* (−1.95 to −1.44)				
90–94	1.30* (0.99 to 1.61)	1990–1995	−0.19 (−0.88 to 0.51)	1995–2000	3.99* (3.01 to 4.98)	2000–2005	−4.44* (−5.3 to −3.58)	2005–2010	9.99* (9.04 to 10.96)	2010–2015	−1.99* (−2.89 to −1.08)	2015–2021	1.09* (0.55 to 1.64)		
≥95	2.52* (1.83 to 3.22)	1990–2003	0.97* (0.74 to 1.21)	2003–2006	6.08* (1.62 to 10.73)	2006–2009	12.13* (7.44 to 17.02)	2009–2013	6.09* (3.81 to 8.41)	2013–2017	0.9 (−1.26 to 3.11)	2017–2021	−3.59* (−5.04 to −2.13)		

AAPC, average annual percent change; APC, annual percent change; LCDMDS, liver cancer due to metabolic dysfunction-associated steatohepatitis.

* Indicates that AAPC or APC significantly different from 0 (2-sided *P* < 0.05).

**Table 3.** Joinpoint regression analysis on the death rates of LCDMDS in Chinese population aged 55 yr or older, 1990–2021

Group	Total study period AAPC (%, 95% CI)	Trend 1		Trend 2		Trend 3		Trend 4		Trend 5		Trend 6		Trend 7	
		Years	APC (%, 95% CI)	Years	APC (%, 95% CI)	Years	APC (%, 95% CI)	Years	APC (%, 95% CI)	Years	APC (%, 95% CI)	Years	APC (%, 95% CI)	Years	APC (%, 95% CI)
Total	0.42 (−0.09 to 0.93)	1990–1994	−0.45 (−2.36 to 1.49)	1994–2001	2.39* (1.39 to 3.41)	2001–2006	−3.03* (−4.71 to −1.32)	2006–2017	2.39* (1.92 to 2.86)	2017–2021	−3.06* (−5.18 to −0.9)				
Sex															
Male	0.60* (0.18 to 1.03)	1990–1995	−0.17 (−0.79 to 0.45)	1995–2001	3.38* (2.77 to 4)	2001–2006	−2.78* (−3.54 to −2.01)	2006–2009	2.4 (−0.01 to 4.86)	2009–2012	0.24 (−2.06 to 2.6)	2012–2015	4.20* (1.43 to 7.05)	2015–2021	−1.09* (−1.64 to −0.55)
Female	0.38 (−0.09 to 0.85)	1990–2001	1.24* (0.77 to 1.72)	2001–2006	−3.21* (−4.97 to −1.41)	2006–2017	2.99* (2.49 to 3.5)	2017–2021	−4.42* (−6.78 to −2)						
Age															
55–59	−0.64* (−1.03 to −0.25)	1990–1998	−1.30* (−1.85 to −0.75)	1998–2002	3.04* (0.47 to 5.68)	2002–2015	−1.86* (−2.13 to −1.58)	2015–2021	0.49 (−0.44 to 1.43)						
60-64	−0.22* (−0.69 to 0.25)	1990–2001	0.11 (−0.15 to 0.37)	2001–2005	−4.01* (−5.78 to −2.2)	2005–2012	1.39* (0.74 to 2.05)	2012–2015	4.47* (0.44 to 8.67)	2015–2021	−2.37* (−3.1 to −1.64)				
65–69	−0.07* (−0.38 to 0.23)	1990–1993	−0.89 (−1.98 to 0.21)	1993–2001	1.29* (1.02 to 1.57)	2001–2006	−4.42* (−5.02 to −3.83)	2006–2012	1.77* (1.31 to 2.23)	2012–2015	7.98* (5.77 to 10.23)	2015–2019	−2.16* (−3.2 to −1.11)	2019–2021	−5.87* (−8.14 to −3.53)
70–74	0.09 (−0.36 to 0.54)	1990–1997	0.81* (0.42 to 1.2)	1997–2001	2.64* (1.17 to 4.13)	2001–2006	−4.48* (−5.3 to −3.65)	2006–2009	2.79* (0.06 to 5.6)	2009–2012	−1.02 (−3.71 to 1.75)	2012–2016	2.50* (1.02 to 4)	2016–2021	−1.08* (−1.77 to −0.38)
75–79	0.34 (−0.03 to 0.72)	1990–1993	−1.49 (−3.47 to 0.54)	1993–2001	3.08* (2.55 to 3.61)	2001–2005	−2.57* (−4.32 to −0.8)	2005–2018	0.81* (0.58 to 1.03)	2018–2021	−2.94* (−4.96 to −0.88)				
80–84	1.61* (0.05 to 3.20)	1990–1995	−3.55* (−6.03 to −1)	1995–2002	6.06* (3.98 to 8.17)	2002–2006	−1.73 (−6.86 to 3.68)	2006–2009	11.59 (−1.82 to 26.83)	2009–2014	4.72* (1.14 to 8.42)	2014–2021	−3.16* (−4.66 to −1.63)		
85–89	0.76* (0.08 to 1.44)	1990–2006	0.06 (−0.47 to 0.59)	2006–2014	5.75* (3.81 to 7.73)	2014–2021	−3.12* (−5.05 to −1.15)								
90–94	1.17* (0.15 to 2.20)	1990–2001	1.27* (0.41 to 2.13)	2001–2006	−3.88* (−7.37 to −0.25)	2006–2010	10.78* (4.21 to 17.77)	2010–2021	0.09 (−0.76 to 0.94)						
≥95	2.49* (1.60 to 3.39)	1990–2007	1.30* (1 to 1.59)	2007–2010	14.80* (5.87 to 24.48)	2010–2016	4.52* (2.62 to 6.45)	2016–2021	−2.67* (−4.57 to −0.73)						

AAPC, average annual percent change; APC, annual percent change; LCDMDS, liver cancer due to metabolic dysfunction-associated steatohepatitis.

* Indicates that AAPC or APC significantly different from 0 (2-sided *P* < 0.05).

### The age-period-cohort analysis of the incidence rate and death rate of LCDMDS

**Figure 2.**
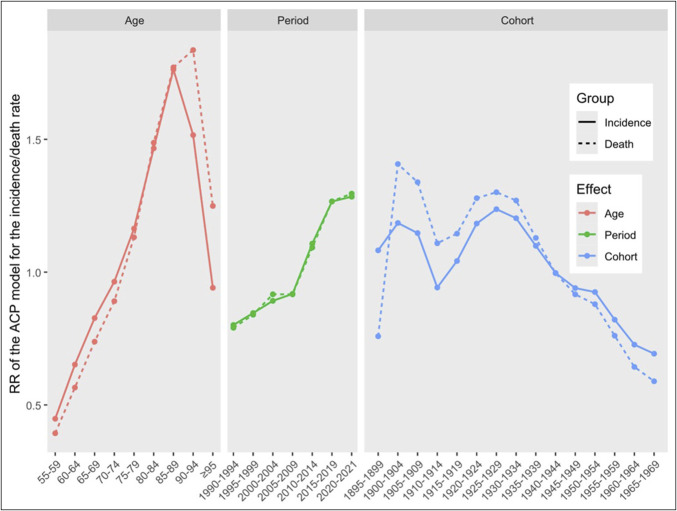
RRs of age, period, and cohort effects on LCDMDS incidence rates and death rates in Chinese population aged 55 years or older during 1990–2021. The red, green and blue dot-line represents the age, period, and cohort effects for LCDMDS. LCDMDS, liver cancer due to metabolic dysfunction-associated steatohepatitis; RR, relative risk.

Age-period- cohort analysis was conducted on the incidence and death rates of LCDMDS in the Chinese population aged 55 years and older to explore the impacts of age, period, and cohort factors on the risk of LCDMDS incidence and death (Table 4). The age effect results revealed that the risk of both LCDMDS incidence and death first increased and then decreased with age, showing an inverted “U”-shaped pattern. The incidence risk was lowest in the 55–59 years age group and highest in the 85–89 years age group, with RRs ranging from 0.45 to 1.76. The death risk was lowest in the 55–59 years age group and highest in the 90–94 years age group, with RRs ranging from 0.39 to 1.84. The period effect results indicated that the risk of LCDMDS incidence and death exhibited a monotonically increasing trend over time, and the 2 trends were highly similar. The incidence and death risks were lowest in the 1990–1994 period (RR for incidence = 0.80, RR for death = 0.79) and highest in the 2020–2021 period (RR for incidence = 1.28, RR for death = 1.30). The cohort effect results demonstrated that the risk of LCDMDS incidence and death presented an “M”-shaped change with the development of the birth cohort. The 1925–1929 birth cohort had the highest incidence risk (RR = 1.24), and the incidence risk of subsequent cohorts gradually decreased. The 1900–1904 birth cohort had the highest death risk (RR = 1.41), followed by a fluctuating decline. The RRs of the incidence and death rates are presented in Figure 2.

**Table 4.** APC model analysis of LCDMDS incidence rates and death rates in Chinese population aged 55 yr or older, 1990–2021

Effect	Incidence rate				Death rate			
	RR	SE	Z	*P*	RR	SE	Z	*P*
Age								
55–59	0.45	0.09	−8.71	<0.001	0.39	0.09	−10.18	<0.001
60–64	0.65	0.07	−6.15	<0.001	0.57	0.07	−8.27	<0.001
65–69	0.83	0.05	−3.99	<0.001	0.74	0.05	−6.45	<0.001
70–74	0.96	0.03	−1.28	0.20	0.89	0.03	−4.24	<0.001
75–79	1.16	0.02	7.47	<0.001	1.13	0.02	6.93	<0.001
80–84	1.47	0.03	11.47	<0.001	1.49	0.03	12.91	<0.001
85–89	1.76	0.05	10.41	<0.001	1.77	0.05	10.99	<0.001
90–94	1.52	0.08	5.13	<0.001	1.84	0.08	7.91	<0.001
≥95	0.94	0.14	−0.44	0.66	1.25	0.12	1.81	0.07
Period								
1990–1994	0.80	0.07	−3.14	<0.001	0.79	0.07	−3.38	<0.001
1995–1999	0.85	0.05	−3.42	<0.001	0.84	0.05	−3.63	<0.001
2000–2004	0.89	0.03	−4.16	<0.001	0.92	0.03	−3.23	<0.001
2005–2009	0.92	0.01	−6.17	<0.001	0.92	0.01	−6.39	<0.001
2010–2014	1.11	0.03	3.93	<0.001	1.09	0.03	3.45	<0.001
2015–2019	1.27	0.05	4.99	<0.001	1.27	0.05	5.06	<0.001
2020–2021	1.28	0.07	3.57	<0.001	1.30	0.07	3.74	<0.001
Cohort								
1895–1899	1.08	0.79	0.10	0.92	0.76	0.78	−0.35	0.72
1900–1904	1.18	0.29	0.59	0.56	1.41	0.26	1.31	0.19
1905–1909	1.15	0.20	0.68	0.50	1.34	0.20	1.49	0.14
1910–1914	0.94	0.17	−0.35	0.72	1.11	0.17	0.62	0.53
1915–1919	1.04	0.14	0.29	0.78	1.14	0.14	0.96	0.34
1920–1924	1.18	0.12	1.42	0.16	1.28	0.12	2.11	0.04
1925–1929	1.24	0.10	2.25	0.03	1.30	0.09	2.82	0.01
1930–1934	1.20	0.07	2.58	0.01	1.27	0.07	3.39	<0.001
1935–1939	1.10	0.05	1.93	0.05	1.13	0.05	2.52	0.01
1940–1944	1.00	0.03	−0.11	0.91	1.00	0.03	−0.14	0.89
1945–1949	0.94	0.02	−4.13	<0.001	0.92	0.02	−5.80	<0.001
1950–1954	0.92	0.03	−2.94	<0.001	0.88	0.03	−4.80	<0.001
1955–1959	0.82	0.05	−4.10	<0.001	0.76	0.05	−5.71	<0.001
1960–1964	0.73	0.07	−4.46	<0.001	0.64	0.07	−6.20	<0.001
1965–1969	0.69	0.10	−3.76	<0.001	0.59	0.10	−5.44	<0.001

LCDMDS, liver cancer due to metabolic dysfunction-associated steatohepatitis; RR, relative risk.

### The ARIMA analysis of LCDMDS from 1990 to 2030

**Figure 3.**
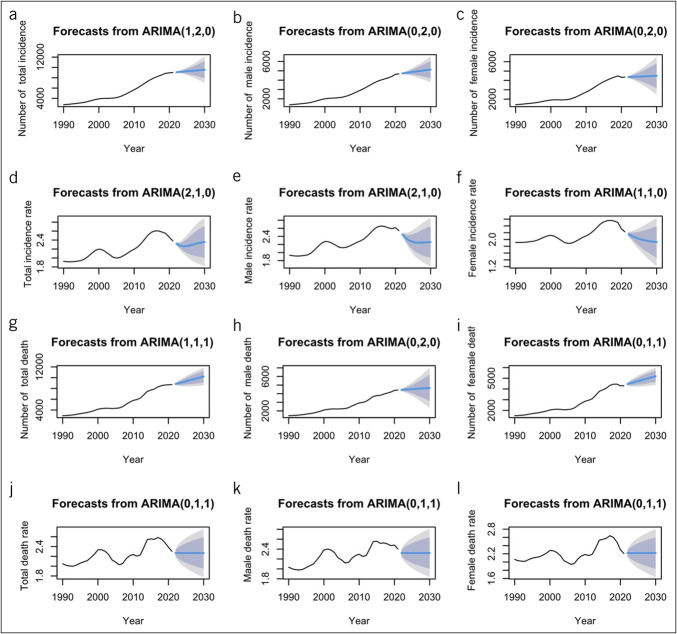
Predicted incidence and death trends of LCDMDS in the Chinese population aged 55 years or older over the next 9 years (2022-30). (**a**) Predicted number of total incidence cases; (**b**) Predicted number of male incidence cases; (**c**) Predicted number of female incidence cases; (**d**) Predicted total incidence rates; (**e**) Predicted male incidence rates; (**f**) Predicted female incidence rates; (**g**) Predicted number of total death cases; (**h**) Predicted number of male death cases; (**i**) Predicted number of female death cases; (**j**) Predicted total death rates; (**k**) Predicted male death rates; (**l**) Predicted female death rates. Black lines represent the true trend of LCDMDS during 1990–2021; blue and shaded regions represent the predicted trend (with 80% and 95% confidence intervals). LCDMDS, liver cancer due to metabolic dysfunction-associated steatohepatitis.

The incidence and death data of LCDMDS among the Chinese population aged 55 years and older from 1990 to 2021 were transformed into time series. If the time series were nonstationary, differencing was conducted to make them stationary, and the Ljung-Box test was performed on the residual series. The prediction models all represented white noise series, indicating no autocorrelation among the observed values. These models demonstrated good fitting capabilities and could accurately predict the future disease burden of LCDMDS in China. According to the prediction results (Figure 3), from 2022 to 2030, the number of LCDMDS incidence cases in China will continue to grow. By 2030, the number of incidence cases will increase to 9,539 (95% UI: 6,980–12,098), a 5.78% increase compared with 2021. The number of deaths will also keep rising. By 2030, the number of deaths will reach 10,223 (95% UI: 8,566–11,879), a 17.22% increase relative to 2021. The incidence rate may experience fluctuating changes with a slight upward trend, whereas the death rate will remain stable (Table 5).

**Table 5.** ARIMA analysis of LCDMDS in Chinese population aged 55 yr or older from 2022 to 2030

Year	Number of total incidence (95% UI)	Number of male incidence (95% UI)	Number of female incidence (95% UI)	Total incidence rate (95% CI)	Male incidence rate (95% CI)	Female incidence rate (95% CI)	Number of total death (95% UI)	Number of male death (95% UI)	Number of female death (95% UI)	Total death rate (95% CI)	Male death rate (95% CI)	Female death rate (95% CI)
2022	9,079 (8,979–9,179)	4,723 (4,641–4,806)	4,362 (4,240–4,483)	2.32 (2.28–2.35)	2.44 (2.39–2.50)	2.16 (2.08–2.23)	8,839 (8,618–9,061)	4,442 (4,302–4,582)	4,462 (4,311–4,614)	2.27 (2.18–2.36)	2.32 (2.23–2.41)	2.22 (2.11–2.32)
2023	9,138 (8,876–9,399)	4,773 (4,589–4,958)	4,378 (4,107–4,650)	2.28 (2.19–2.37)	2.36 (2.23–2.48)	2.10 (1.95–2.25)	8,994 (8,484–9,503)	4,471 (4,158–4,784)	4,553 (4,233–4,873)	2.27 (2.07–2.46)	2.32 (2.13–2.51)	2.22 (1.99–2.45)
2024	9,196 (8,718–9,673)	4,824 (4,515–5,133)	4,395 (3,940–4,849)	2.26 (2.10–2.43)	2.29 (2.08–2.51)	2.05 (1.82–2.29)	9,163 (8,412–9,913)	4,500 (3,977–5,023)	4,644 (4,218–5,070)	2.27 (2.01–2.53)	2.32 (2.07–2.58)	2.22 (1.91–2.53)
2025	9,253 (8,514–9,992)	4,874 (4,422–5,326)	4,411 (3,746–5,076)	2.27 (2.02–2.51)	2.26 (1.96–2.56)	2.02 (1.70–2.34)	9,337 (8,384–10,289)	4,529 (3,763–5,295)	4,734 (4,224–5,245)	2.27 (1.96–2.58)	2.32 (2.01–2.63)	2.22 (1.85–2.59)
2026	9,310 (8,271–10,350)	4,925 (4,313–5,537)	4,428 (3,527–5,328)	2.28 (1.96–2.61)	2.25 (1.87–2.62)	1.99 (1.59–2.39)	9,513 (8,387–10,639)	4,557 (3,520–5,594)	4,825 (4,242–5,409)	2.27 (1.91–2.62)	2.32 (1.97–2.67)	2.22 (1.8–2.64)
2027	9,368 (7,992–10,743)	4,975 (4,188–5,762)	4,444 (3,286–5,603)	2.31 (1.91–2.70)	2.24 (1.80–2.69)	1.97 (1.49–2.45)	9,690 (8,412–10,969)	4,586 (3,252–5,920)	4,916 (4,268–5,564)	2.27 (1.88–2.66)	2.32 (1.93–2.71)	2.22 (1.75–2.69)
2028	9,425 (7,683–11,167)	5,025 (4,049–6,002)	4,461 (3,024–5,898)	2.33 (1.88–2.78)	2.25 (1.75–2.75)	1.95 (1.39–2.51)	9,868 (8,452–11,283)	4,615 (2,960–6,270)	5,007 (4,300–5,713)	2.27 (1.84–2.70)	2.32 (1.90–2.74)	2.22 (1.71–2.73)
2029	9,482 (7,345–11,619)	5,076 (3,897–6,254)	4,477 (2,743–6,212)	2.34 (1.85–2.84)	2.25 (1.70–2.80)	1.94 (1.30–2.57)	10,045 (8,505–11,586)	4,644 (2,646–6,641)	5,097 (4,337–5,858)	2.27 (1.81–2.73)	2.32 (1.87–2.78)	2.22 (1.67–2.77)
2030	9,539 (6,980–12,098)	5,126 (3,733–6,519)	4,494 (2,443–6,544)	2.36 (1.82–2.89)	2.26 (1.66–2.86)	1.92 (1.21–2.63)	10,223 (8,566–11,879)	4,672 (2,312–7,033)	5,188 (4,377–5,999)	2.27 (1.78–2.76)	2.32 (1.84–2.81)	2.22 (1.63–2.80)

CI, confidence interval; LCDMDS, liver cancer due to metabolic dysfunction-associated steatohepatitis; UI, uncertainty interval.

## DISCUSSION

This study describes and predicts the epidemiological trends in the burden of disease for LCDMDS among people aged 55 years and older in China with the help of data from the GBD Study (GBD) 2021, resulting in several important findings. The study showed that from 1990 to 2021, the number of incidence, incidence rate, deaths, and death rate of LCDMDS among people aged 55 years and older in China showed a significant upward trend. Among them, the number of incidence increased by 226.38% and the number of deaths increased by 196.43%, which indicated that the development of the disease outpaced the growth of the population. Joinpoint regression showed that the incidence rate of LCDMDS increased by 0.72% per year on average, but the death rate did not increase significantly. Age-period-cohort analysis results indicated that the risk of incidence and death in LCDMDS demonstrated a pattern of first increasing and then decreasing with age. Meanwhile, it gradually escalated over time and declined with the development of the birth cohort. ARIMA prediction analysis revealed that, in the forthcoming period, the number of incidences and deaths of LCDMDS among the Chinese population aged 55 years and older will keep rising, signifying that the disease burden of LCDMDS will remain extremely heavy.

In the context of rapidly accelerating global aging, investigating the disease burden of LCDMDS among middle-aged and elderly individuals is of great significance. MASH and its associated hepatocellular carcinoma pose an increasingly severe health threat to the middle-aged and elderly people. With economic development, lifestyle changes, such as the prevalence of high-sugar and high-fat diets and reduced physical activity, have led to more frequent metabolic disorders such as obesity and diabetes, driving up the global incidence of MASH (30). For instance, in the United States, the proportion of obese people has been rising, and the MASH incidence increased from around 10% in 1990 to 25% in 2019 (31), significantly increasing the medical burden. Macro-data show that in 1990, the proportions of morbidity, mortality, and disability-adjusted life years for LCDMDS were 4.74%, 5.30%, and 4.25%, respectively, and by 2019, these had increased by 43.5%, 35.3%, and 49.4%, respectively, clearly demonstrating the growing disease burden. In China, improved living standards have altered lifestyles (15). MASH has a 20%–30% detection rate in the examined population, with a trend toward younger age groups (32,33). Unhealthy habits cause many to fall ill, indicating MASH has become a common liver disease affecting public health. Given the development trend of MASH and its serious threat to the health of middle-aged and elderly individuals, researching the disease burden of LCDMDS in Chinese people aged older than 55 years is imperative. This study not only offers data support for understanding the pathogenesis and epidemiological trends of LCDMDS but also provides a scientific basis for formulating prevention and control strategies, allocating medical resources, and enhancing the health and quality of life of the middle-aged and elderly.

There exists a disparity in the burden of LCDMDS among Chinese individuals aged 55 years or older, with the burden on men being slightly heavier than that on women. The growth curves of the number of incidences and deaths of LCDMDS in men and women were highly overlapping, showing no conspicuous gender differences. Nevertheless, since 1996, the incidence rate of LCDMDS in men has been gradually higher than that in women, and the average annual growth rate of incidence rate is also higher in men (0.94% vs 0.51%). Regarding death rate, although the death rate of LCDMDS in women is comparable with that in men, the mean annual growth rate of mortality is higher in men, and this difference is statistically significant. Previous research has also revealed that the burden of LCDMDS is on the rise in both genders. However, the incidence rates in women are close to those in men (8,14), whereas the death rates are lower, with a female-to -male mortality ratio of 0.9 for LCDMDS. From 1990 to 2019, the overall burden of liver cancer was higher in men, despite the fact that the incidence and mortality rates in women were close to those in men (15). There are 3 primary reasons for the gender differences. First, high androgen levels in men regulate fatty acid transporter proteins, promoting hepatic fat accumulation and increasing the risk of MASH development (34,35). By contrast, high premenopausal estrogen levels in women suppress inflammation and apoptosis, reducing the risk (36). Although the risk increases after menopause, it remains lower than that in men. Second, men are more likely to have bad habits such as smoking, excessive alcohol consumption, and lack of exercise. Smoking disrupts liver metabolism (37), alcohol damages the liver, and lack of exercise causes obesity, which aggravates the liver burden and contributes to MASH development, resulting in higher morbidity and mortality in men (38,39). Finally, men have higher levels of hepatic cytochrome P450 enzymes and other metabolic enzymes (40). When exposed to harmful substances, they produce more oxidative stress products that damage hepatocytes (41). In addition, they respond more robustly to inflammatory signals and are prone to persistent inflammation and fibrosis during the inflammatory phase (42), increasing the risk of morbidity and mortality. In summary, there are gender differences in the incidence rate and death rate of LCDMDS in the Chinese population aged 55 years or older, albeit the differences are minor.

We further used the age-period-cohort analysis approach to analyze the effects of age, period, and cohort factors on LCDMDS incidence rate and death rate. In the age-effect curve, a significant rise in the risk of LCDMDS incidence and death was observed with age increase (15,43), reaching its peak at the age of 85–89 years. As age advances, the body's metabolic function deteriorates gradually. The liver's capacity for fat metabolism and detoxification reduces, and the long-term accumulation of environmental factors and adverse lifestyle impacts persists (44). This makes MASH more likely to occur and progress to liver cancer, significantly increasing the risk of LCDMDS incidence and death (45).After the age of 85–89 years, the risk of LCDMDS incidence and death gradually drops. We believe this may be due to the following combined factors. First, after the age of 85 years, overall body functions weaken severely, and hepatocytes gradually enter a senescent state with reduced cell-division ability (7,46). This may no longer sustain continuous tumor growth. For example, the body's nutrient absorption and metabolism are severely restricted, making it hard for tumor cells to get nutrients, thus limiting tumor deterioration and reducing the LCDMDS morbidity and mortality risk (47). Second, the elderly often have multiple chronic diseases such as cardiovascular and respiratory diseases. When the age exceeds 85 years, these chronic diseases may become dominant causes of death, competing with LCDMDS (48,49).Therefore, the future focus of LCDMDS prevention and control in China should be on the elderly.

The period effect demonstrated that the RRs of LCDMDS incidence rate and death rate increased over time. This indicates that, after excluding the effects of age and cohort factors, the risk of LCDMDS incidence and death in Chinese individuals aged 55 years or older is on the rise, fully validating our research hypothesis. The linear growth trend is evident, with the risk of LCDMDS incidence increasing by 60% and the risk of death increasing by 64.56% in 2020–2021 compared with 1990–1994. Several factors contribute to this concerning outcome. First, China's population growth and the deepening aging process have increased the proportion of the elderly, leading to a rise in the incidence and mortality of LCDMDS. Second, the prevalence of unhealthy lifestyles, such as high-calorie diets and lack of exercise, has increased the incidence of metabolic diseases such as obesity, indirectly augmenting the risk of LCDMDS morbidity and mortality (50). In addition, certain episodic events can affect LCDMDS. For instance, the LCDMDS incidence was 2.13 and the mortality was 2.11 in 2019, which increased to 2.38 and 2.30 in 2021, respectively. The COVID-19 pandemic might have accelerated the progression of MASH, contributing in part to the increase in LCDMDS incidence and mortality (51,52). In the context of lifestyle changes and population aging, the burden of LCDMDS among Chinese middle-aged and elderly individuals is growing and demands attention.

The cohort effect indicated that the RRs of LCDMDS incidence rate and death rate were declining for birth cohorts starting from 1925 to 1929. This implies that the risk of LCDMDS incidence and death decreases as the birth cohort advances, which is encouraging. For instance, compared with the 1925–1929 birth cohort, the 1965–1969 birth cohort had a 79.71% reduction in the risk of LCDMDS development and a 120.34% reduction in the risk of death. We believe multiple factors have jointly contributed to this positive change. In the first place, with social development, the Chinese population has received more health education, reducing the MASH incidence base. In the second place, China's medical technology has been advancing, with increasingly sophisticated disease diagnosis and screening methods. This allows for earlier detection of liver diseases such as LCDMDS and timely intervention and treatment (53). Effective control of chronic diseases such as hypertension and hyperlipidemia has also indirectly lowered the LCDMDS risk. Improvements in environmental hygiene, including better drinking water quality and less exposure to environmental pollution, have reduced liver damage from environmental factors (54). Moreover, the implementation of public health policies, such as stricter food hygiene regulations, has helped decrease the intake of harmful chemicals and pathogens, safeguarding liver health (55,56), and thus reducing the risk of LCDMDS incidence and death. Continuous efforts to increase intervention are still essential to further reduce the risk of LCDMDS incidence and mortality in China in the future.

Monitoring disease prevalence and predicting trends are crucial for disease prevention and control. Despite the encouraging cohort-effect results, the ARIMA model predicts that the number of LCDMDS incidences and deaths in the Chinese population aged 55 years or older will exhibit a linear growth trend. By 2030, these numbers are expected to reach 9,539 and 10,223 cases, respectively, representing a 5% increase compared with 2021. The incidence and death rates will remain stable at 2.3/100,000 and 2.2/100,000, respectively. This indicates a future rise in the LCDMDS burden in China, highlighting the need for vigilance. East Asia, including China, is one of the world's most aging regions, and population aging is the main factor contributing to the heavy future LCDMDS burden in China (57). Proactively addressing aging is essential for reducing the LCDMDS burden among Chinese middle-aged and elderly people. It is imperative to take immediate action to enhance LCDMDS prevention and treatment in this population, alleviate the disease burden in the elderly, and achieve a healthy - aging society.

Between 1990 and 2021, the number of incidence, incidence rate, deaths, and death rate of LCDMDS among Chinese individuals aged 55 years or older increased remarkably, and the future burden will remain substantial. The accelerated global aging process and lifestyle changes have augmented the threat posed by LCDMDS. Regarding gender differences, the burden of LCDMDS is slightly heavier in men than in women. Age-period-cohort studies have indicated that the risk of incidence and death first rises and then declines with age, increases over time, and decreases with the development of the birth cohort. Moreover, the number of incidences and deaths is projected to grow in the future. Therefore, prevention and control efforts should be targeted at the elderly. Increasing interventions, coping with aging, and establishing a liver-health support system are crucial steps to reduce the risk of LCDMDS morbidity and mortality.

## CONFLICTS OF INTEREST

**Guarantor of the article:** Qiaomei Li, PhD and Yang Shen, PhD.

**Specific author contributions:** X.Y.: Writing—original draft, Methodology, Investigation, Formal analysis. X.Y.: Writing—review & editing, Formal analysis. H.W.: Methodology, Investigation, Formal analysis. Q.L. & Y.S.: Validation, Supervision, Methodology, Investigation, Formal analysis, Conceptualization.

**Financial support:** This study was funded by the Leapfrog Program of the Third Affiliated Hospital of Naval Medical University (TF2024YSSH06). The funders did not intervene in the design, methodology, data collection, analysis and preparation of the manuscript.

**Potential competing interests:** None to report.

**Data availability:** The data that support the findings of this study are available from the corresponding author upon reasonable request.

**Study Highlights**

**WHAT IS KNOWN**

✓ LCDMDS burden is increasing.
✓ Lack of long-term studies on high-risk group.

**WHAT IS NEW HERE**

✓ Analyze trends with Global Burden of Disease 2021 data.
✓ Evaluate age, period, cohort effects.
✓ Predict burden with autoregressive integrated moving average model.

## ABBREVIATIONS:

AAPC: average APC
APC: annual percentage change
AR: autoregressive
ARIMA: autoregressive integrated moving average
DALY: disability-adjusted life year
GBD: Global Burden of Disease
LCDMDS: liver cancer due to metabolic dysfunction-associated steatohepatitis
MA: moving average
MASH: metabolic dysfunction-associated steatohepatitis
RR: relative risk
UI: uncertainty interval
